# MTHFD2-mediated redox homeostasis promotes gastric cancer progression under hypoxic conditions

**DOI:** 10.1080/13510002.2024.2345455

**Published:** 2024-05-09

**Authors:** Hai-Yu Mo, Ruo-Bing Wang, Meng-Yao Ma, Yi Zhang, Xin-Yu Li, Wang-Rong Wen, Yi Han, Tian Tian

**Affiliations:** 1 Department of Medical Biochemistry and Molecular Biology, School of Medicine, Jinan University, Guangzhou, People’s Republic of China; 2 Clinical Laboratory, The Affiliated Shunde Hospital of Jinan University, Foshan, People’s Republic of China; 3 State Key Laboratory of Oncology in South China, Guangdong Provincial Clinical Research Center for Cancer, Sun Yat-sen University, Guangzhou, People’s Republic of China

**Keywords:** Gastric cancer, methylene tetrahydrofolate dehydrogenase 2 (MTHFD2), redox metabolism, NADPH, reactive oxygen species (ROS)

## Abstract

**Objectives:**

Cancer cells undergo metabolic reprogramming to adapt to high oxidative stress, but little is known about how metabolic remodeling enables gastric cancer cells to survive stress associated with aberrant reactive oxygen species (ROS) production. Here, we aimed to identify the key metabolic enzymes that protect gastric cancer (GC) cells from oxidative stress.

**Methods:**

ROS level was detected by DCFH-DA probes. Multiple cell biological studies were performed to identify the underlying mechanisms. Furthermore, cell-based xenograft and patient-derived xenograft (PDX) model were performed to evaluate the role of MTHFD2 in vivo.

**Results:**

We found that overexpression of MTHFD2, but not MTHFD1, is associated with reduced overall and disease-free survival in gastric cancer. In addition, MTHFD2 knockdown reduces the cellular NADPH/NADP+ ratio, colony formation and mitochondrial function, increases cellular ROS and cleaved PARP levels and induces in cell death under hypoxia, a hallmark of solid cancers and a common inducer of oxidative stress. Moreover, genetic or pharmacological inhibition of MTHFD2 reduces tumor burden in both tumor cell lines and patient-derived xenograft-based models.

**Discussion:**

our study highlights the crucial role of MTHFD2 in redox regulation and tumor progression, demonstrating the therapeutic potential of targeting MTHFD2.

## GRAPHICAL ABSTRACT

**Figure:**
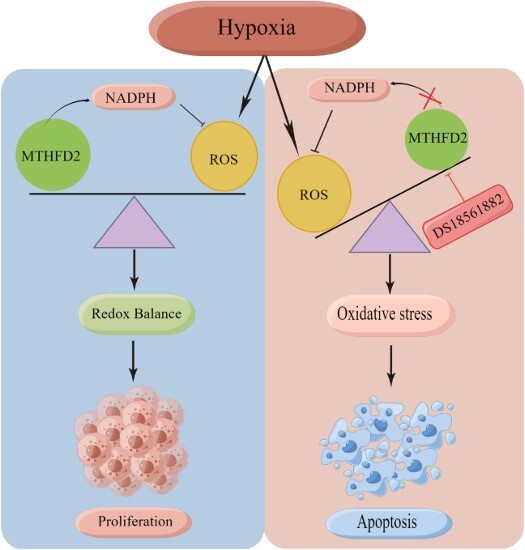

## 1. Introduction

Gastric carcinogenesis (GC) remains a leading cause of cancer-related death worldwide. Greater than one million new cases of GC were diagnosed in 2020. GC ranks fifth in incidence and fourth in mortality among cancers globally [1]. Mounting evidence suggests that many types of cancer cells, including GC cells, exhibit increased levels of reactive oxygen species (ROS) compared with their normal counterparts [2–5]. Therefore, sustaining ROS homeostasis is critical for cancer cell growth and survival. Cancer cells maintain ROS homeostasis by counteracting ROS accumulation with ROS elimination through ROS-scavenging systems, which largely depend on the production of NADPH [6, 7]. Several metabolic pathways contribute to the generation of NADPH, such as the pentose phosphate pathway, glutamine metabolism, fatty acid oxidation and one-carbon folate metabolism [2, 8–10]. Although the role of one-carbon folate metabolism in NADPH production and ROS homeostasis has been studied deeply, little is known about whether the one-carbon folate metabolism is related to GC prognosis and progression.

The folate metabolism pathway contributes to cell proliferation by producing one-carbon formyl groups for de novo purine and thymidine synthesis [9, 11]. In addition, this pathway also plays a key role in generating the reducing power NADPH [12]. In this pathway, both cytoplasmic MTHFD1 and mitochondrial MTHFD2 generate NADPH from NADP^+^, suggesting their critical role in cellular detoxification. Indeed, MTHFD1 overexpression in hepatocellular carcinoma predicts poorer survival and recurrence [13]. In an esophageal cancer model, increased MTHFD1 methylation largely augments the production of NADPH, which resulting in anoikis resistance and distant organ metastasis [14]. In addition, MTHFD2 expression is markedly elevated and correlated with poor survival in lung, colorectal, renal and breast cancer [15–18]. However, whether and how MTHFD1/2 contributes to GC progression has been overlooked and remains largely unknown.

In the present study, we identified that MTHFD2, but not MTHFD1, is overexpressed and associated with poor prognosis in patients with GC. Furthermore, genetic or pharmacological inhibition of MTHFD2 reduces tumor burden in both GC cell lines and patient-derived xenograft-based models. Mechanistically, MTHFD2 suppression diminished the NADPH and GSH contents, resulting in elevated ROS levels and cell death. More importantly, we found that DS18561882, a substrate-based inhibitor of MTHFD2, effectively attenuates MTHFD2-mediated NADPH production and tumor growth. Taken together, our results reveal the critical role of MTHFD2 in redox modulation and provide a potential therapeutic target for GC treatment.

## 2. Materials and methods

### 2.1. Human tissue samples

The GC tissue specimens in this study were collected from the Department of Pathology, Sun Yat-sen University Cancer Center (SYSUCC, Guangzhou, China) after obtaining written informed consent. All patients underwent surgery at the SYSUCC and did not receive preoperative treatment (including radiotherapy); all patients had a clear histopathological and clinical diagnosis of gastric cancer after surgery. Tissues were formalin-fixed in 4% (v/v) buffered formaldehyde and paraffin-embedded for diagnostic purposes. The project was approved by the ethics committee of the SYSUCC, and all procedures were performed in accordance with the seventh version of the Declaration of Helsinki. Overall survival was defined as the time from the date of surgery to the date of death from any cause or latest follow-up, whereas disease-free survival was measured from the date of surgery to the date of confirming recurrence or death from any cause, whichever occurred first.

### 2.2. Cell culture

The human GC cell lines AGS and GES-1 were purchased from the American Type Culture Collection (ATCC, Manassas, VA, U.S.A.), and SNU216, IM95, MKN74, MKN1, KATO Ⅲ, MKN45, NUGC4, Hs746 T, HGC27, SNU668, NUGC3 and FU97 cells were purchased from Cell Bank of Typical Culture Preservation Committee of Chinese Academy of Sciences. All cells were cultured under conditions specified by the supplier. All cells tested negative for mycoplasma contamination and were authenticated based on short tandem repeat fingerprinting before use. The cell lines were maintained in 1640 (Invitrogen; Thermo Fisher Scientific) complete medium supplemented with 10% (v/v) fetal bovine serum (WISENT) and 1% (v/v) penicillin-streptomycin (Gibco by Life Technologies), in a humidified incubator at 37˚C with 5% CO2. Hypoxia was achieved using a Anoxomat™ MARK II system (Advanced Instruments, Norwood, MA, U.S.A.) with a final condition of 1% O_2_, 5% CO_2_, and 94% N_2_.

### 2.3. Cell viability and clonogenic assay

Cell viability was tested as described previously [19]. In brief, GC cells (3 × 10^3^ cells/well) were seeded in 96-well plates overnight, and cell viability was quantified by adding [3-(4,5-dimethylthiazol-2-yl)-5-(3-carboxymethoxyphenyl)-2-(4-sulfophenyl)-2H-tetrazolium, inner salt, MTS] directly to culture wells followed by incubating for 3 h. The MTS is bioreduced by cells into a colored formazan product that is soluble in culture medium. The quantity of formazan product as measured by the amount of 490 nm absorbance is directly proportional to the number of living cells in culture. Cells (800 cells/well) were seeded in 6-well plates and incubated for 10 to 14 days. After colonies were clearly observed, they were fixed with 4% (v/v) formaldehyde and stained with crystal violet (0.5%, w/v). After three rinses with phosphate-buffered saline (PBS), the wells were imaged.

### 2.4. Cell lentiviral-based gene transduction

Short hairpin RNA (shRNA) directed against MTHFD2 was ligated into the pLV12 vector (OBiO Technology, Shanghai, China). Lentiviruses were generated by transfecting lentiviral vector pLV12 together with packaging vector psPAX2 and envelope plasmid pMD2.G into HEK293T cells. Lentiviruses were harvested by collecting cell culture medium and further filtering with 0.22 μm filter. MKN74 and HGC27 cells were infected with lentiviruses and selected with puromycin for one week. Knockdown of target protein was confirmed by immunoblot analysis.

### 2.5. RNA isolation and qPCR analysis

Total RNA was isolated with TRIzol regent (Cat. #15596-08, Life Technologies, Carlsbad, U.S.A.) and then reverse transcribed with a PrimeScript™ RT Master Mix (Cat. #RR036Q, TAKARA, Tokyo, Japan). The resulting complementary DNA was analyzed by qPCR performed with SYBR reagent using the GoTaq qPCR Master Mix (Cat. #A6002, Promega, Madison, U.S.A.). β-Actin was used as the internal control gene, and data were analyzed using the 2^-ΔΔct^ method. The primer s equences were as follows: MTHFD1 (NM_005956.4, forward: 5’-gttgaaggagcaagtacctgg-3’, reverse: 5’-ggtagctgcactaagaaccca-3); MTHFD2 (NM_006636.4, forward: 5’- gatcctggttggcgagaatcc-3’, reverse: 5’-tctggaagaggcaactgaaca-3); β-actin (NM_001101.5, forward: 5’-catgtacgttgctatccaggc-3’, reverse: 5’-ctccttaatgtcacgcacgat-3).

### 2.6. Immunoblotting and IHC analysis

Immunoblotting and IHC analysis were conducted using standard procedures as previously described [20]. Cells were washed twice with ice-cold PBS and lysed in RIPA (#89900, ThermoFisher, Rockford, IL, U.S.A.). The concentration of proteins was normalized using a BCA protein assay (#23225, ThermoFisher, Rockford, IL, U.S.A.). Protein samples were run on a standard SDS-PAGE and transferred to PVDF membranes. Subsequently, the membranes were blotted with specific primary antibodies overnight at 4 ^◦^C. After that, the membranes were incubated with appropriate horseradish peroxidase conjugated secondary antibodies, and the signals were tested by the ECL detection system (#32109, ThermoFisher, Rockford, IL, U.S.A.). The IHC scores were assessed by two independent authors blinded to the patients’ clinicopathological data. We quantitatively scored the tissue sections according to the staining intensity (0, no signal; 1, weak; 2, moderate; and 3, strong) and percentage of positive cells (1, 0%–25%; 2, 26%–50%; 3, 51%–75%; and 4,>75%). We then combined the intensity and proportion scores to obtain a total score as described previously [21]. Specimens with scores ≥4 were classified as high-expression, while those with scores <4 were classified as low-expression. Sections were also stained with hematoxylin and eosin (HE) and TUNEL assay (ab206386, abcam, MA, U.S.A.) according to standard procedures. The following antibodies were used for immunoblotting or IHC analysis: MTHFD2 (1:200 for IHC and 1:1000 for immunoblotting, sc-100750) (Santa Cruz, CA, U.S.A.); cleaved caspase-3 (1:1000, #96664S), c-PARP (1:1000, #9185), β-actin (1:1000, #3700) (Cell Signaling, Beverly, U.S.A.); and Ki67 (1:200, #ZM-0167) (ZSGB-BIO, Beijing, China).

### 2.7. In vivo tumorigenesis and metastasis study

All mouse experiments were performed in accordance with a protocol approved by our Institutional Animal Care and Use Committee. Female BALB/c nude mice (4–5 weeks old) were obtained from Beijing Vital River Laboratory Animal Technology Co., Ltd. (Beijing, China) and housed in a specific pathogen-free animal room. For the in vivo tumorigenesis study, GC cells (3 × 10^6^, in phosphate buffered saline) were subcutaneously injected into the flanks of nude mice. Patient-derived xenograft (PDX) tumors from two patients with gastric metastasis were implanted into the flanks of mice. The dosage of DS18561882 used in vivo was decided as previously described [22, 23]. Tumor size was measured every 4 days using a caliper, and tumor volume was calculated using the standard V = length × width^2^/2. Mice were euthanized when they met the institutional euthanasia criteria for tumor size and overall health condition. The tumors were removed, photographed, weighed and paraffin embedded.

### 2.8. Cell death analysis

For cell death analysis, GC cells were harvested, washed twice and resuspended in 500 μl of PBS plus Annexin V-FITC and propidium iodide (PI) (Cat. #KGA1030-100, KeyGEN, China). The degree of cell death was determined as the percentage of cells positive for Annexin V/PI evaluated by flow cytometry (FACS Calibur, Becton Dickinson) as described previously [24].

### 2.9. Determination of ROS, GSH/GSSG, and NADPH/NADP^+^ levels

Cellular ROS levels were determined by flow cytometry using a CM-H2DCF-DA (#D399) Assay kit (Thermo Fisher Scientific, U.S.A.), as described in previous publications [16]. Lipid ROS levels were measured by flow cytometry using BODIPY 581/591 C11 (#D3861, Thermo Fisher Scientific, U.S.A.) staining. The intracellular levels of GSH/oxidized glutathione (GSSG) and NADPH/NADP^+^ were measured using a GSH/GSSG Assay kit (#V6612) and an NADPH/NADP^+^ Assay kit (#G9081) (Promega, WI) according to the manufacturer’s instructions.

### 2.10. Determination of catalase, SOD and caspase-3 activity

Catalase activity was measured using a catalase activity assay kit (ab83464, abcam, U.S.A.). SOD activity was measured using a superoxide dismutase activity assay kit (ab65354, abcam, U.S.A.). Caspase-3 activity was measured using a caspase-3 assay kit (ab39401, abcam, U.S.A.). All procedures were performed according to the manufacturer’s instructions.

### 2.11. Oxygen consumption rate assay

Cells were pre-treated with hypoxia before oxygen consumption rate assay (OCR). XFe24 Extracellular Flux Analyzer (Seahorse Bioscience, North Billerica, MA, U.S.A.) was used for real-time analysis of OCR according to the manufacturer’s user guide. In brief, cells were seeded overnight in a Seahorse 24-well culture microplate at density 4 × 10^4^ per well. The medium was changed to Seahorse base medium supplemented with 1 mM pyruvate, 2 mM glutamine, and 10 mM glucose on the day of the assay and incubated for 1 h in a CO_2_-free incubator at 37°C prior to the assay. Injections of drugs, oligomycin (1 µM), FCCP (1 µM), and rotenone/antimycin A (0.5µM) were loaded onto ports A, B, C, and D respectively. Results were normalized to cell number.

### 2.12. Statistical analysis

Statistical analyses were performed with GraphPad version 8.0. Student’s *t* test was used for comparison of the significant differences between two groups. Matched groups (three or more) were compared using one-way analysis of variance ANOVA and Tukey’s multiple comparisons test. Survival curves were plotted using the Kaplan–Meier method and compared using the log-rank test. The parameters with P less than .05 in univariate analyses were included in the multivariable Cox analysis. Gaussian distribution was assessed using D'Agostino-Pearson test. *P* < 0.05 was considered statistically significant. All statistical tests were two-sided.

## 3. Results

### 3.1. MTHFD2 is highly expressed and predicts poor prognosis in GC

**Figure 1.**
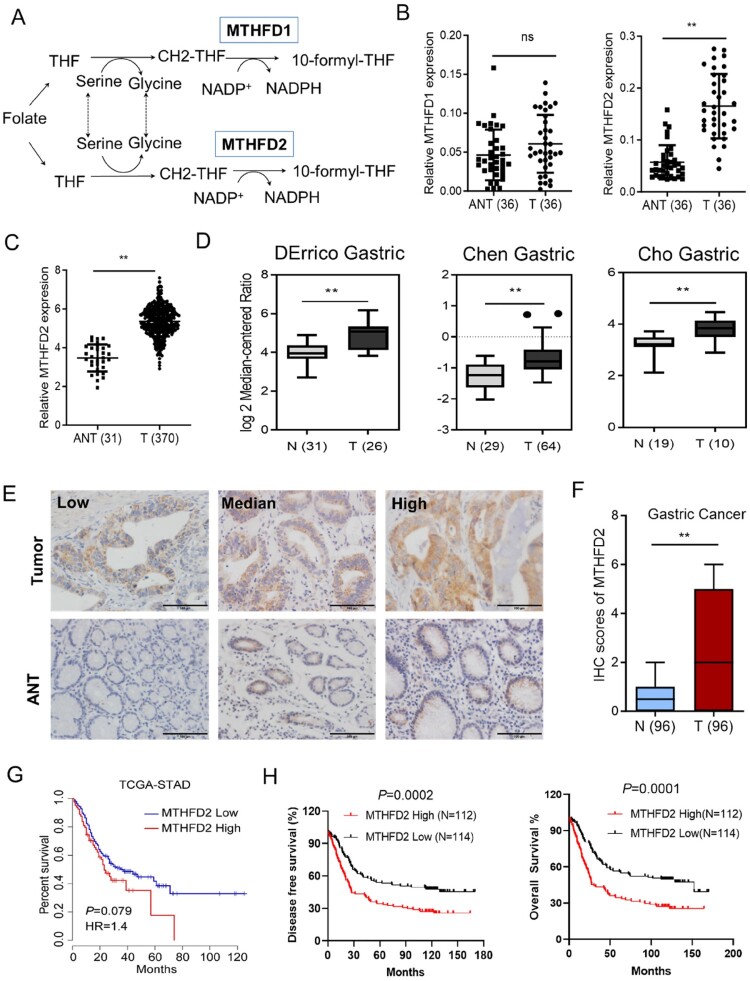
MTHFD2 is highly expressed and predicts poor prognosis in GC. (A) Schematic representation of the function of MTHFD1/2 in folate metabolism. (B) Quantitative polymerase chain reaction (qPCR) analysis of MTHFD1/2 expression in 36 paired GC tissues. (C–D) MTHFD2 expression profiling in multiple GC microarray datasets from the Oncomine (C) and TCGA (D) databases. (E) Immunohistochemical analyses of 96 human GC specimens and their paired adjacent normal gastric tissues were performed. MTHFD2 in tumor tissues is higher than in adjacent tissues. Representative staining images are shown (scale bar = 100 μm). (F) Immunohistochemical staining scores of MTHFD2 in paired primary GC tumor and paired normal tissues. (G-H) Kaplan–Meier analysis of 5-year survival (G), overall survival and disease-free survival (H) for GC patients with low vs. high MTHFD2 expression. MTHFD1/2 = methylene tetrahydrofolate dehydrogenase 1/2; N = normal tissues; ANT = adjacent normal tissues; T = tumor. Data are presented as mean ± SD, Student’s *t* test (non-parametric comparisons for B and D, parametric comparison for C and F) or log-rank test (G-H). ns, not significant, **P < 0.01.

Both MTHFD1 and MTHFD2 participate in folate metabolism (Figure 1(A)). We first evaluated the MTHFD1/2 mRNA levels in cancerous and matched paracancerous tissues in GC patients using quantitative polymerase chain reaction (qPCR) analysis. The results revealed that MTHFD2, but not MTHFD1, was overexpressed in tumor tissues vs. matched paracancerous tissues (Figure 1(B)). Furthermore, the overexpression of MTHFD2 in GC was also supported by other microarray datasets of GC available from Oncomine and The Cancer Genome Atlas database (TCGA) (Figure 1(C,D)). Consistently, immunohistochemical analysis confirmed that the MTHFD2 expression level was statistically increased in cancerous tissues compared with paracancerous tissues, suggesting that MTHFD2 was aberrantly upregulated in GC (Figure 1(E,F)). Additionally, Kaplan-Meier survival analysis showed that patients with high MTHFD2 expression levels had a shorter percent survival, overall survival (HR = 1.99, 95% CI = 1.42 to 2.79, *p* = 0.0002) and disease-free survival (HR = 1.87, 95% CI = 1.34 to 2.62, *p* = 0.0001) (Figure 1(G,H)). Taken together, our results suggest that MTHFD2 is a potential prognostic biomarker and a promising target for GC treatment.

### 3.2. MTHFD2 suppression inhibits GC cell proliferation

**Figure 2.**
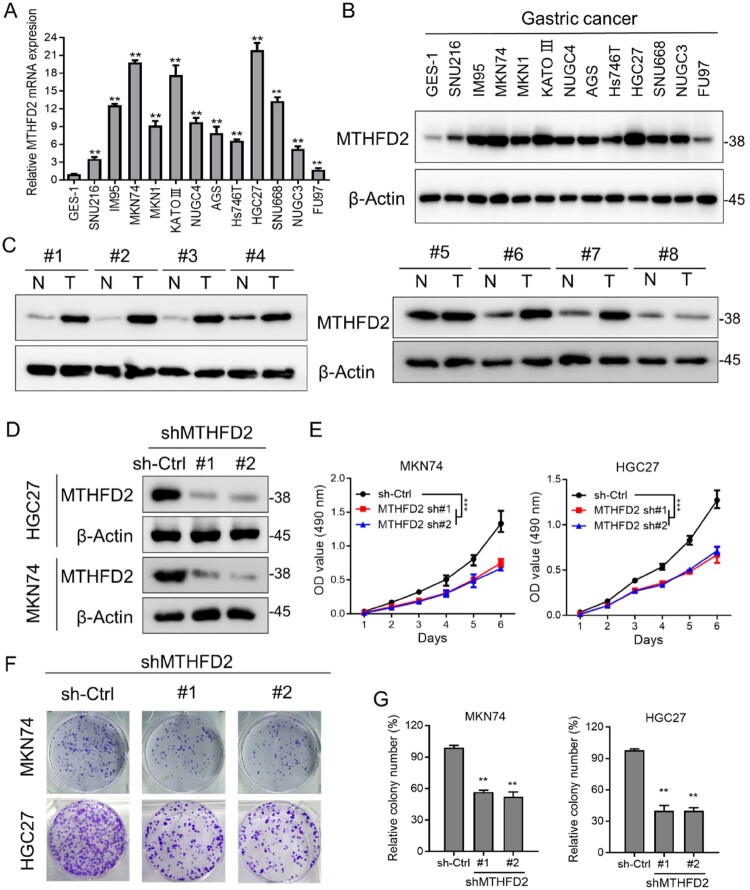
MTHFD2 suppression inhibits GC cell proliferation. (A–B) qPCR and immunoblotting analysis of MTHFD2 expression levels in gastric cancer cell lines. (C) Immunoblotting analysis of MTHFD2 protein levels in paired GC and normal tissues. (D) Immunoblotting evaluating the knockdown efficiency of MTHFD2 with two unique shRNAs (#1, #2) in HGC27 and MKN74 cells. (E) Proliferation of sh-Control and sh-MTHFD2 MKN74 and HGC27 cells. (F-G) Colony formation assays in MKN74 and HGC27 cells after knockdown of MTHFD2. β-Actin was included as a loading control. Data are presented as mean ± SD, one-way ANOVA (non-parametric comparisons for A and G) or two-way ANOVA (E). ns, not significant, **P < 0.01.

Next, we analyzed the pattern of MTHFD2 expression in various GC cell lines. As shown in Figure 2(A), MTHFD2 mRNA levels were dramatically elevated in all detected GC cell lines compared with nontumorigenic cells (GES1) (Figure 2(A)). Consistently, the MTHFD2 protein levels were notably increased in various GC cell lines and GC patient samples (Figure 2(B)). To clarify the role of MTHFD2 in GC cells, we first stably knocked down MTHFD2 in MKN74 and HGC27 cell lines, and the knockdown efficiency was confirmed using immunoblotting analysis (Figure 2(D)). We next examined the effect of MTHFD2 suppression on cell proliferation. As expected, MTHFD2 knockdown reduced MKN74 and HGC27 cell proliferation (Figure 2(E)). To test the long-term impact of MTHFD2 knockdown on cell renewal and proliferation, a colony formation assay was performed. As shown in Figure 2(F,G), MTHFD2 knockdown significantly reduced MKN74 and HGC27 cell colony formation. These results suggest that MTHFD2 promotes GC cell proliferation in vitro.

### 3.3. MTHFD2 knockdown impairs NADPH homeostasis and accelerates GC cell death under hypoxia

**Figure 3.**
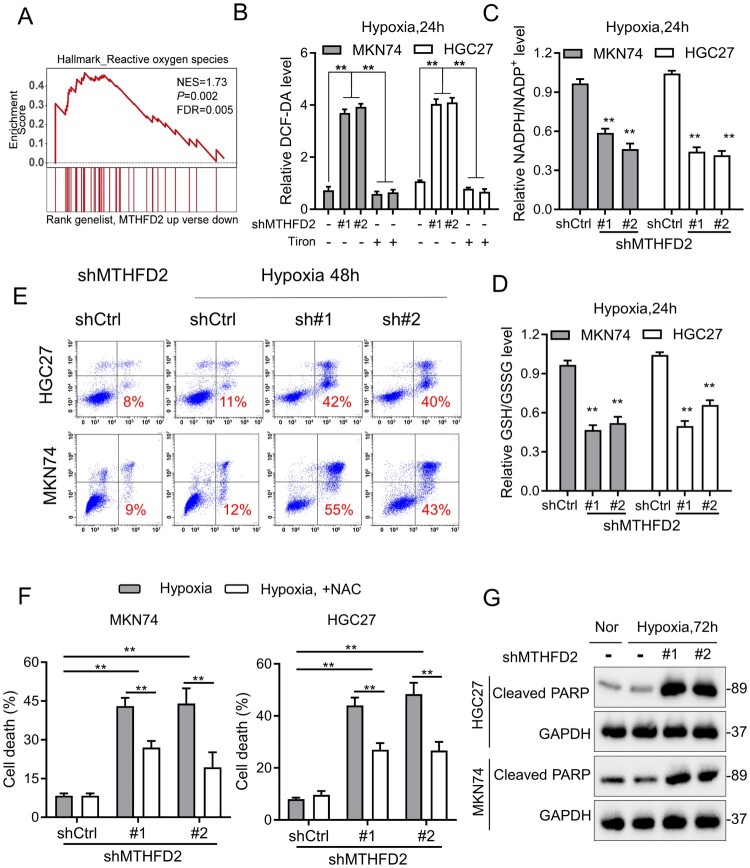
MTHFD2 knockdown impairs NADPH homeostasis and accelerates GC cell death under hypoxia. (A) Gene set enrichment score and distribution of ROS-related genes along the rank of MTHFD2 up vs. MTHFD2 down available from The Cancer Genome Atlas GC database. (B–D) Cellular ROS (B), NADPH/NADP^+^ (C) and GSH/GSSG (D) levels were measured in indicated GC cells under hypoxic conditions for 24 h. (E) Cell death was measured by Annexin-V/PI assays in the indicated GC cells under normal and hypoxic conditions for 48 h (red numbers indicate the subpopulation of cells positive for Annexin V/PI). (F) Quantification of cell death in the indicated cells cultured under hypoxic conditions for 72 h (with or without 5 mM N-acetyl-L-cysteine). (G) Immunoblotting analysis of cleaved poly ADP-ribose polymerase (PARP) in the indicated GC cells. GAPDH was included as a loading control. Nor = normoxia. Data are presented as mean ± SD, one-way ANOVA (non-parametric comparisons). ns, not significant, **P < 0.01.

We next explored the possible mechanisms by which MTHFD2 contributed to GC cell proliferation. As growing evidence indicates the fundamental roles of redox regulation in tumor growth, we hypothesized that MTHFD2-mediated NADPH homeostasis may promote GC tumorigenesis. Expectedly, gene set enrichment analysis (GSEA) revealed the enrichment of ROS-related signature in gastric tumors with high expression of MTHFD2 (normalized enrichment score = 1.73, *p* = 0.002) (Figure 3(A)), demonstrating its critical role in redox homeostasis. Hypoxia occurs when tumor growth exceeds the capacity of available vasculature during tumor progression. Previous research has shown that hypoxic exposure obviously induces mitochondrial and cellular ROS generation [25, 26]; thus, we postulated that MTHFD2 was required to maintain redox balance by producing NADPH under hypoxia. Indeed, knockdown of MTHFD2 in MKN74 and HGC27 cells caused notable increases in cellular ROS in spite of lipid ROS remained unchanged (Figure 3(B), Supplementary Figure 1A). In contrast, a reduction in NADPH/NADP^+^ ratio, GSH/GSSG ratio, catalase activity and oxygen consumption rate (OCR) were noted in MTHFD2-depleted cells under conditions of hypoxia (Figure 3(C,D), Supplementary Figure 1B–E). We further performed cell death assay to test the protective effect of MTHFD2 in response to the cytotoxic effect of hypoxia. Although there was no obvious difference in the control groups under normal or hypoxic conditions, MTHFD2 knockdown dramatically induced cell death under hypoxia in MKN74 and HGC27 cells. In addition, we also observed obvious expression of cleaved PARP (c-PARP) in GC cells depleted of MTHFD2 under hypoxia for 72 h. Interestingly, hypoxia-induced cell death in MTHFD2-depleted cells could be rescued by the antioxidant N-acetyl-L-cysteine (NAC), indicating that the increased cell death caused by MTHFD2 repression was due to elevated ROS (Figure 3(E–G)). In summary, these data indicate that MTHFD2 is essential for redox homeostasis maintenance and promoting GC cell survival under conditions of hypoxia.

### 3.4. MTHFD2 knockdown suppresses GC tumorigenesis in vivo

**Figure 4.**
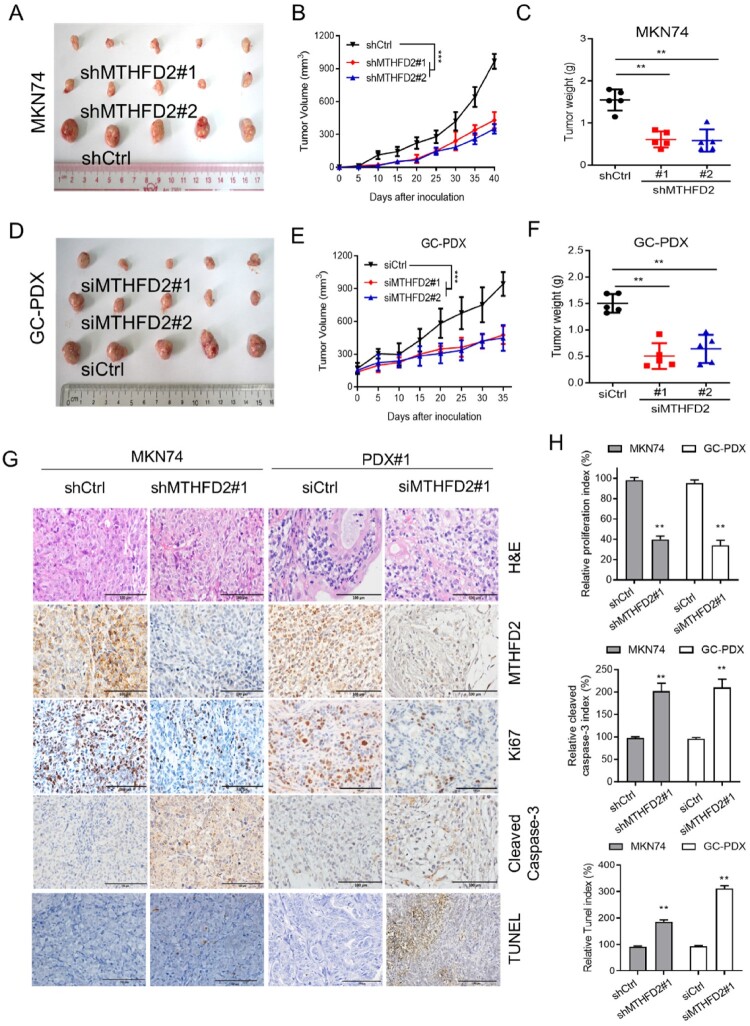
MTHFD2 knockdown suppresses GC tumorigenesis in vivo. (A–C) A xenograft model was established in nude mice subcutaneously implanted with MTHFD2-knockdown and control MKN74 cells (*N* = 5). Tumor images are presented (A). Tumor volumes were calculated (B) Tumor weights were obtained (C). (D–F) Patient-derived xenograft (PDX) tumors were implanted into the flanks of mice. Mice were treated with either PBS or si-MTHFD2. Tumor images are shown (D). Tumor volumes were calculated (E). Tumor weights were obtained (F). (G) Paraffin-embedded tumor sections derived from the indicated group were stained with hematoxylin and eosin (HE) or MTHFD2, Ki67 and cleaved caspase 3 antibodies or TUNEL assay. Ki67 levels are lower, whereas cleaved caspase 3 and TUNEL levels are higher, in tumor tissues with MTHFD2 knockdown. Representative staining images are shown (scale bar = 100 μm). H. The proliferation index (Ki67 staining), apoptotic index (cleaved caspase 3) and TUNEL index in tumor sections were quantified. Data are presented as mean ± SD, one-way ANOVA (parametric comparisons, C and F), Student’s t test (non-parametric comparison, H) or two-way ANOVA (B and E). ns, not significant, **P < 0.01.

To evaluate whether MTHFD2 promotes GC progression in vivo, we conducted cell-based xenograft model by subcutaneously injecting equal amounts of MTHFD2 knockdown or control MKN74 or HGC27 cells into BALB/c nude mice. As expected, MTHFD2 knockdown significantly suppressed tumor growth in vivo, as evidenced by a slower growth rate and reduced tumor weight (Figure 4(A–C), Supplementary Figure 2A–C). We next performed patient-derived xenograft (PDX) model which has been used as a more realistic experimental and preclinical model. We further confirmed the antitumor effect of MTHFD2-targeting siRNA in mice bearing PDX tumors. As expected, MTHFD2-targeting siRNA treatment dramatically suppressed tumor growth in the PDX model compared with the control group (Figure 4(D–F)). Furthermore, tumor samples generated from MTHFD2 knockdown HGC27 and MKN74 cells as well as PDX-induced tumors suggested limited cell proliferation ability and reinforced cell death compared with control group, as determined by Ki67, caspase 3 activity, cleaved caspase 3 and TUNEL staining, respectively (Figure 4(G–H), Supplementary Figure 2D–E). Taken together, our results highlight the criitical role of MTHFD2 in promoting GC progression in vivo.

### 3.5. DS18561882 reduces NADPH production and the growth of GC cells in vitro

**Figure 5.**
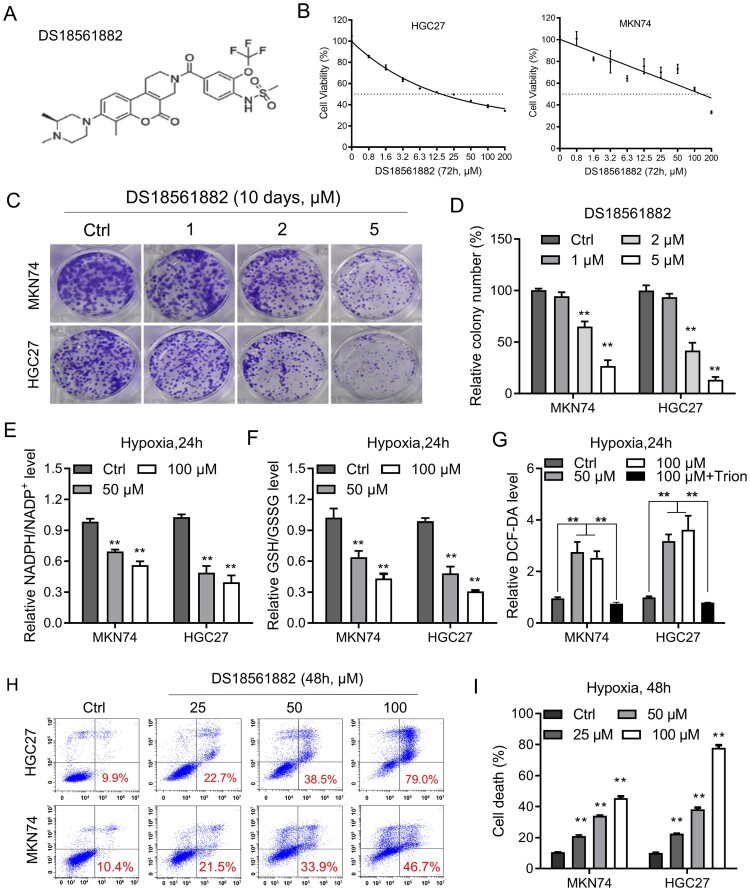
DS18561882 reduces NADPH production and GC cell growth in vitro. (A) Chemical structure of DS18561882. (B) The viability of the indicated GC cells treated with DS18561882 for 72 h was determined by MTS assay. (C–D) Colony formation assays in MKN74 and HGC27 cells treated with DS18561882. (E-G) Cellular NADPH/NADP^+^ (E), GSH/GSSG (F) and ROS (G) levels were measured in the indicated GC cells treated with DS18561882 under conditions of hypoxia for 24 h. (H–I) Cell death was measured using Annexin-V/PI assays in the indicated GC cells treated with DS18561882 under normal or hypoxic conditions for 48 h (red numbers indicate the subpopulation of cells positive for Annexin V/PI). Data are presented as mean ± SD, one-way ANOVA (non-parametric comparisons). ns, not significant, **P < 0.01.

DS18561882 is a substrate-based inhibitor of MTHFD2 and exhibits antitumor activity in a mouse xenograft model [23]. As shown in Figure 5(B), DS18561882 treatment resulted in a dose-dependent enhancement of the reduction of MKN74 and HGC27 cell viability (Figure 5(B)). Consistent with MTHFD2 knockdown, MTHFD2 inhibition by DS18561882 dramatically suppressed colony formation in these two cell lines (Figure 5(C–D)). We next tested the effect of DS18561882 treatment in GC cells under hypoxia. As expected, DS18561882 treatment resulted in significant reductions in NADPH/NADP + and GSH/GSSG ratios but accumulation of cellular ROS (Figure 5(E–G), *p* < 0.01). Accordingly, GC cells treated with DS18561882 exhibited dose-dependent cell death under hypoxia for 48 h (Figure 5(H,I)). Collectively, our findings indicate that pharmacological inhibition of MTHFD2 by DS18561882 effectively impaired NADPH homeostasis and GC cell proliferation.

### 3.6. DS18561882 exerts antitumor effects against GC in vivo

**Figure 6.**
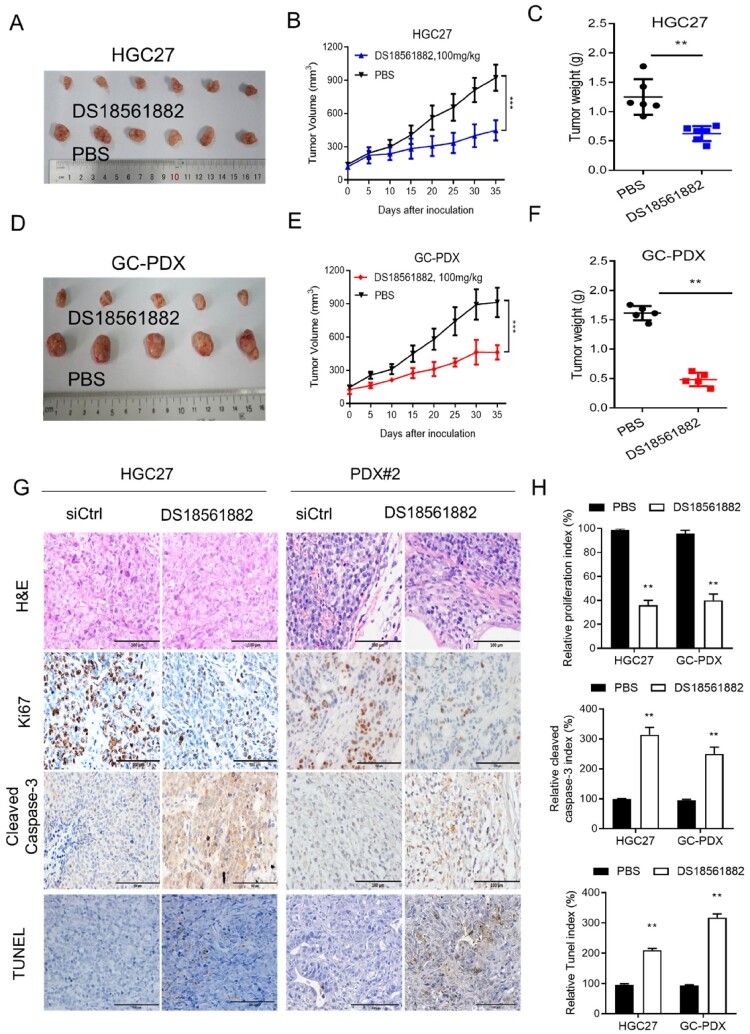
DS18561882 exerts antitumor effects against GC in vivo. (A–C) A xenograft model was established in nude mice subcutaneously implanted with MTHFD2-knockdown and control HGC27 cells (*N* = 6). Tumor images are shown (A). Tumor volumes were calculated (B). Tumor weights were obtained (C). (D–F) Patient-derived xenograft (PDX) tumors were implanted into the flanks of mice. Mice were treated either with PBS or DS18561882. Tumor images are shown (D). Tumor volumes were calculated (E). Tumor weights were obtained (F). (G) Paraffin-embedded tumor sections derived from the indicated group were stained with hematoxylin and eosin (HE) or MTHFD2, Ki67, and cleaved caspase 3 antibodies or TUNEL assay. Ki67 levels are lower, whereas cleaved caspase 3 levels are higher, in tumor tissues with MTHFD2 inhibition. Representative staining images are shown (scale bar = 100 μm). H. The proliferation index (Ki67 staining), apoptotic index (cleaved caspase 3) and TUNEL index in tumor sections were quantified. Data are presented as mean ± SD, Student’s t test (parametric comparisons for C and F, non-parametric comparison for H) or two-way ANOVA (B and E). ns, not significant, **P < 0.01.

To test whether DS18561882 suppresses GC progression in vivo, we conducted cell-based xenograft model by subcutaneously injecting equal amounts of log-phase HGC27 cells into BALB/c nude mice. As expected, the control group treated with PBS exhibited a large tumor burden in all six mice (Figure 6(A)). In contrast, the DS18561882 treatment group experienced a greater than 50% reduction in tumor burden, as evidenced by the significantly mitigated tumor growth and reduced tumor weight (Figure 6(B–C)). We further confirmed the antitumor effect of DS18561882 in vivo using a PDX model. Consistently, DS18561882 treatment induced a 70% reduction in tumor burden (Figure 6(D–F)). Notably, treatment with DS18561882 was well tolerated which was evident by the normal and physically active phenomenon as well as no significant weight loss was observed (Supplementary Figure 2A–B). IHC staining revealed that DS18561882 treatment caused limited proliferation indices and enhanced cell death compared to the control treatment (Figure 6(G–H) and supplementary Figure 2C). Collectively, our findings indicate that DS18561882 possesses antitumor activity against GC and warrants further clinical investigation for GC treatment.

## 4. Discussion

Metabolic reprogramming is a hallmark of malignancy [27, 28]. In particular, management of redox homeostasis is indispensable to sustaining normal cellular functions and assuring cell survival [5, 29, 30]. Disorders in ROS metabolism and redox signaling pathways are often appeared in cancer cells and participate in tumor progression [31, 32]. However, although the role of folate metabolism in NADPH generation and redox balance has been acknowledged, the mechanisms by which folate metabolism exerts its biological functions and whether it is related to GC prognosis remain largely unknown. In this study, we found that MTHFD2 is necessary for the production of NADPH under states of oxidative stress, such as hypoxia. Moreover, genetic or pharmacological inhibition of MTHFD2 reduces tumor burden in both tumor cell lines and PDX-based models. Altogether, our study highlights the critical role of MTHFD2 in redox homeostasis and tumor progression, demonstrating the therapeutic potential of targeting MTHFD2.

The folate metabolism pathway contributes to cell proliferation and survival by producing one-carbon formyl groups for various cellular processes, including de novo purine and thymidine synthesis [11, 33, 34]. Additionally, the folate cycle produces NADPH for use in maintaining redox homeostasis which is necessary for cellular detoxification [12]. In support of this notion, growing evidence has revealed that MTHFD1 and MTHFD2 levels are notably upregulated and correlated with poor prognosis across various cancer types [35–40]. Although the enzymes of folate metabolism are expressed in both the mitochondria and cytosol, mitochondria are the dominant region in the production of formate in most cells for use in cytosolic nucleotide synthesis [41]. Indeed, we show here that MTHFD2, but not MTHFD1, was overexpressed in GC cell lines and patient tissues. Electron leakage from the mitochondrial respiratory chain is the major source of ROS in cancer cells [42]. It’s well known that GC cells are under intrinsic oxidative stress and have highly mobilized ROS-scavenging systems, where the availability of the mitochondrial NADPH pool seems to be a crucial component [43, 44]. MTHFD2 is an important enzyme that catalyzes the generation of mitochondrial NADPH to maintain cellular redox homeostasis [45]. Thus, it is not surprising that inhibition of MTHFD2 caused a significant reduction in NADPH, resulting in abnormal ROS accumulation and ultimately cellular oxidative stress. Although ferroptosis is highly correlated with NADPH, MTHFD2 inhibition mildly affected lipid ROS accumulation. Since lipid peroxidation is produced by iron-dependent peroxidation of polyunsaturated fatty acids (PUFA), both iron and PUFA account for the production and accumulation of lipid peroxidation. In light of this, MTHFD2 inhibition may infinitesimally influence iron or lipid metabolism which therefore has no effect on lipid peroxidation and ferroptosis. However, MTHFD2 inhibition dramatically induces oxidative stress and cell death. Therefore, the critical role of MTHFD2 in protecting GC cells from oxidative stress makes it highly expressed in tumor tissue. More importantly, our results also suggest that MTHFD2 could represent a potential new therapeutic target and provide a biochemical basis for designing more effective strategies for GC treatment.

Hypoxia is a typical microenvironment feature of almost all solid tumors as a result of the rapid and uncontrolled proliferation of tumors which limits the availability of oxygen [46–48]. Hypoxia can damage both cancer cells and normal cells by inducing severe oxidative stress, however, cancer cells undergo adaptive reprogramming that afford them to survive and even proliferate in this lethal microenvironment [47, 49–51]. In addition, numerous lines of evidence suggest that hypoxia contributes to the cancer stem cell phenotype, invasion, and resistance to chemo- and immunotherapies [49, 52]. Therefore, it is important to elucidate the mechanisms by which hypoxia affects tumor progression, which can lead to the development of novel therapeutic methods. Intriguingly, one consequence of hypoxia is the diminished current of electrons that pass through the electron transport chain (ETC) and an increase in electron leakage at the ETC, resulting in the production of ROS [25, 53–55]. Given that MTHFD2 is a crucial enzyme that protects GC cells from elevated ROS, it would then be possible to effectively kill GC cells by suppressing MTHFD2 under hypoxia. Indeed, we show here that inhibition of MTHFD2 by DS18561882 substantially induced ROS accumulation and reduced NADPH and GSH content in GC cells under hypoxia. More importantly, DS18561882 treatment displayed potent antitumor activity in both HGC27 cells and PDX-based tumor models. Together, our data suggest that MTHFD2 could represent a potential therapeutic target.

## 5. Conclusion

Our results suggest that MTHFD2 contributes to redox homeostasis and promotes GC progression in response to hypoxia. Targeting MTHFD2 with its inhibitor DS18561882 exhibits therapeutic efficacy against GC and warrants further clinical investigation for GC treatment.

## Supplementary Material

Supplementary Figure3.tif

Supplementary Figure2.tif

Supplementary Figure1.tif

graphical abstract.tif

## Data Availability

Analyses of the expression of MTHFD2 in GC were supported by the TCGA database (http://www.cbioportal.org/publicportal/) and the Oncomine database (https://www.oncomine.org/). The pathway analysis was performed with gene set enrichment analysis (version 4.0.3).
