# Supplementary figures and images for: MTHFD2-mediated redox homeostasis promotes gastric cancer progression under hypoxic conditions
Source: Redox Rep. 2024 May 9;29(1):2345455. doi: 10.1080/13510002.2024.2345455 (PMC11086033; doi:10.1080/13510002.2024.2345455)

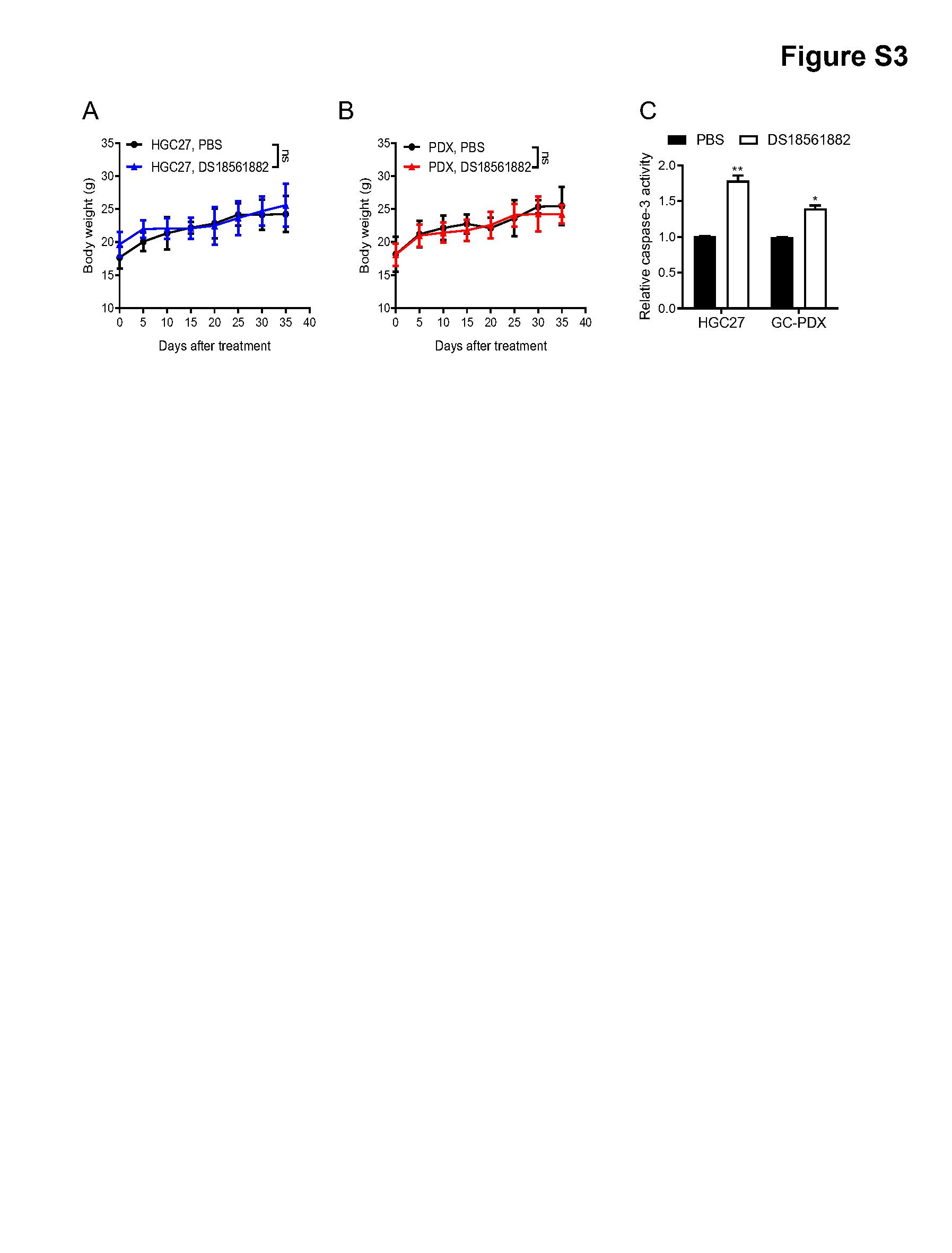

Supplement: Supplementary Figure3.tif [file YRER_A_2345455_SM1382.tif]

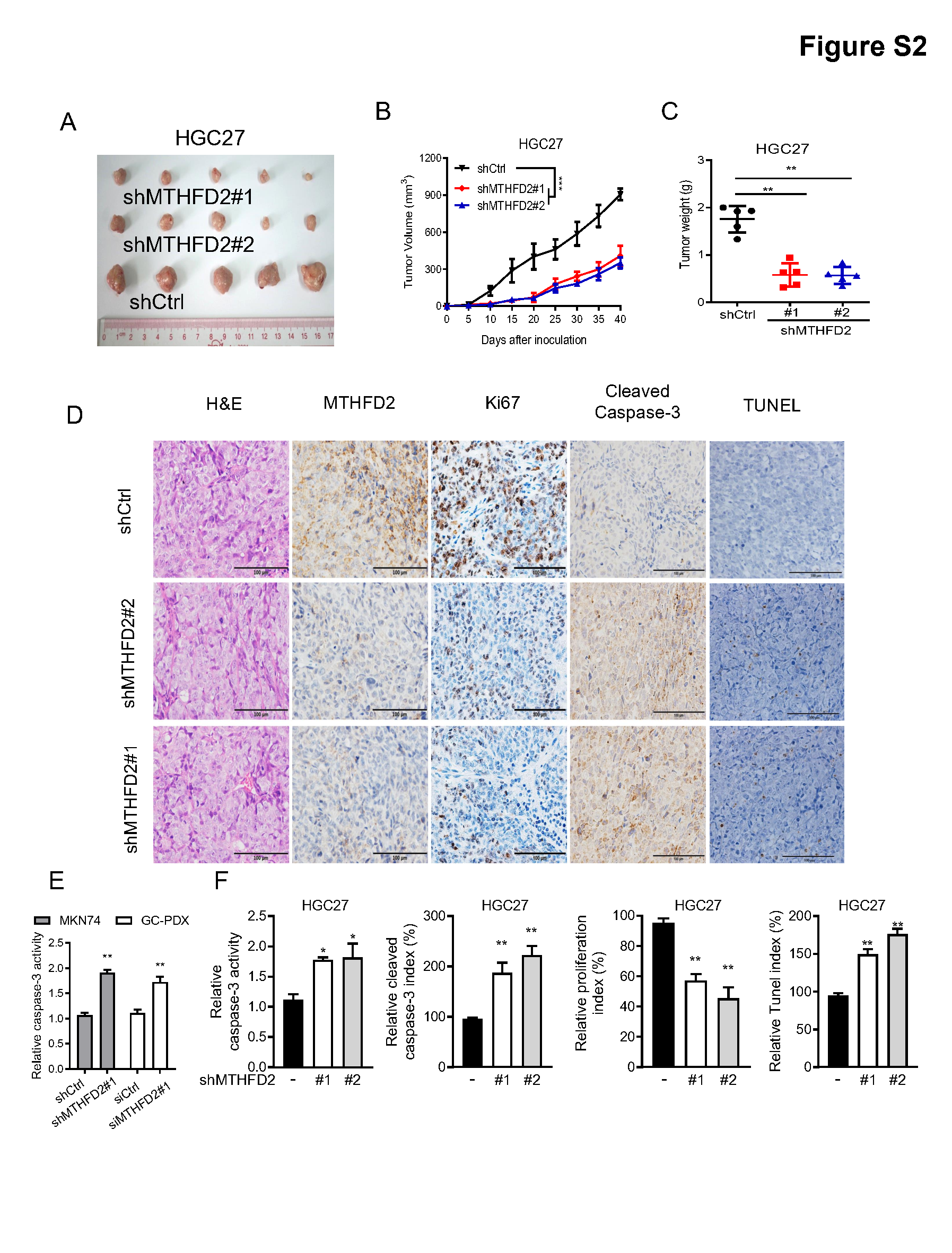

Supplement: Supplementary Figure2.tif [file YRER_A_2345455_SM1381.tif]

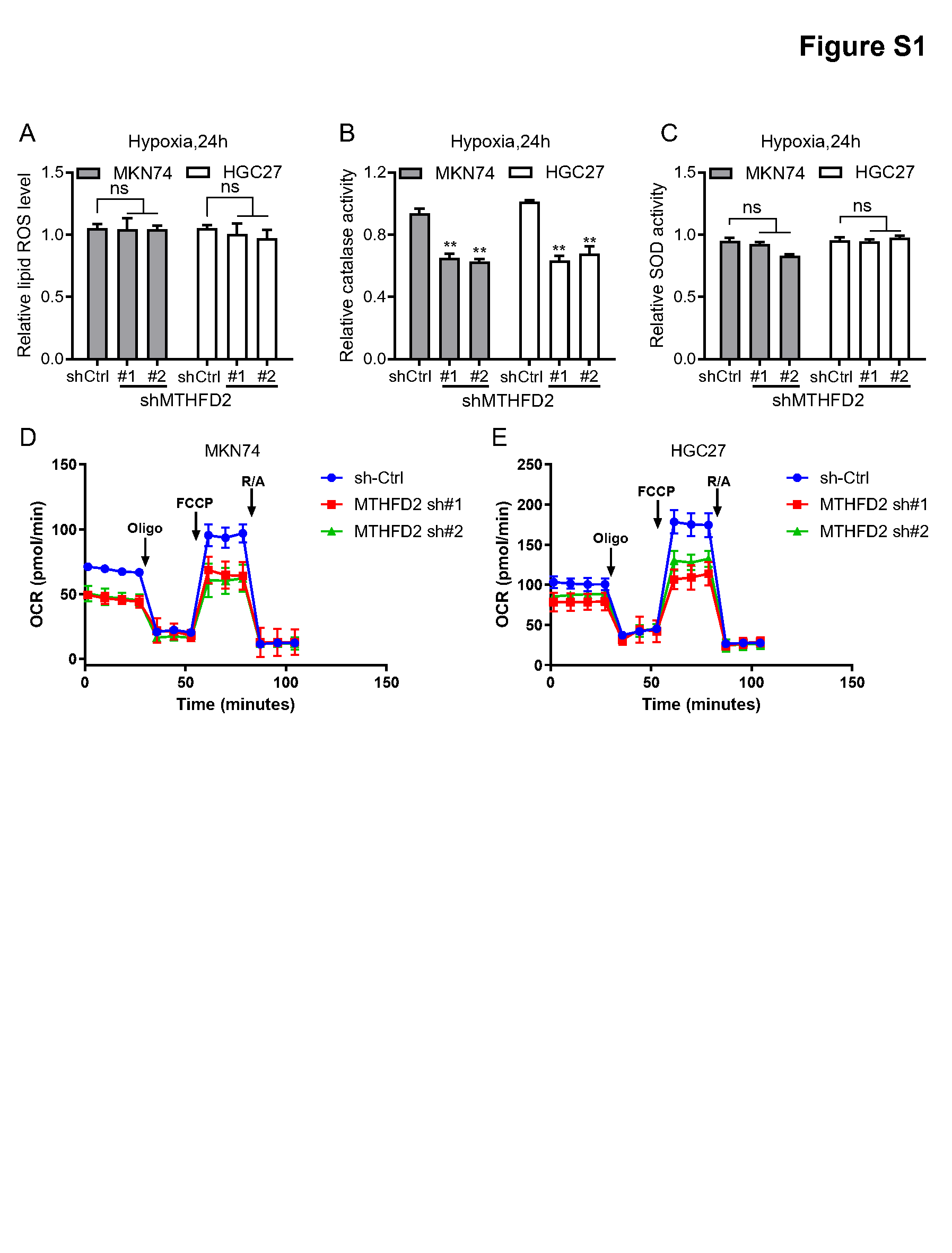

Supplement: Supplementary Figure1.tif [file YRER_A_2345455_SM1380.tif]

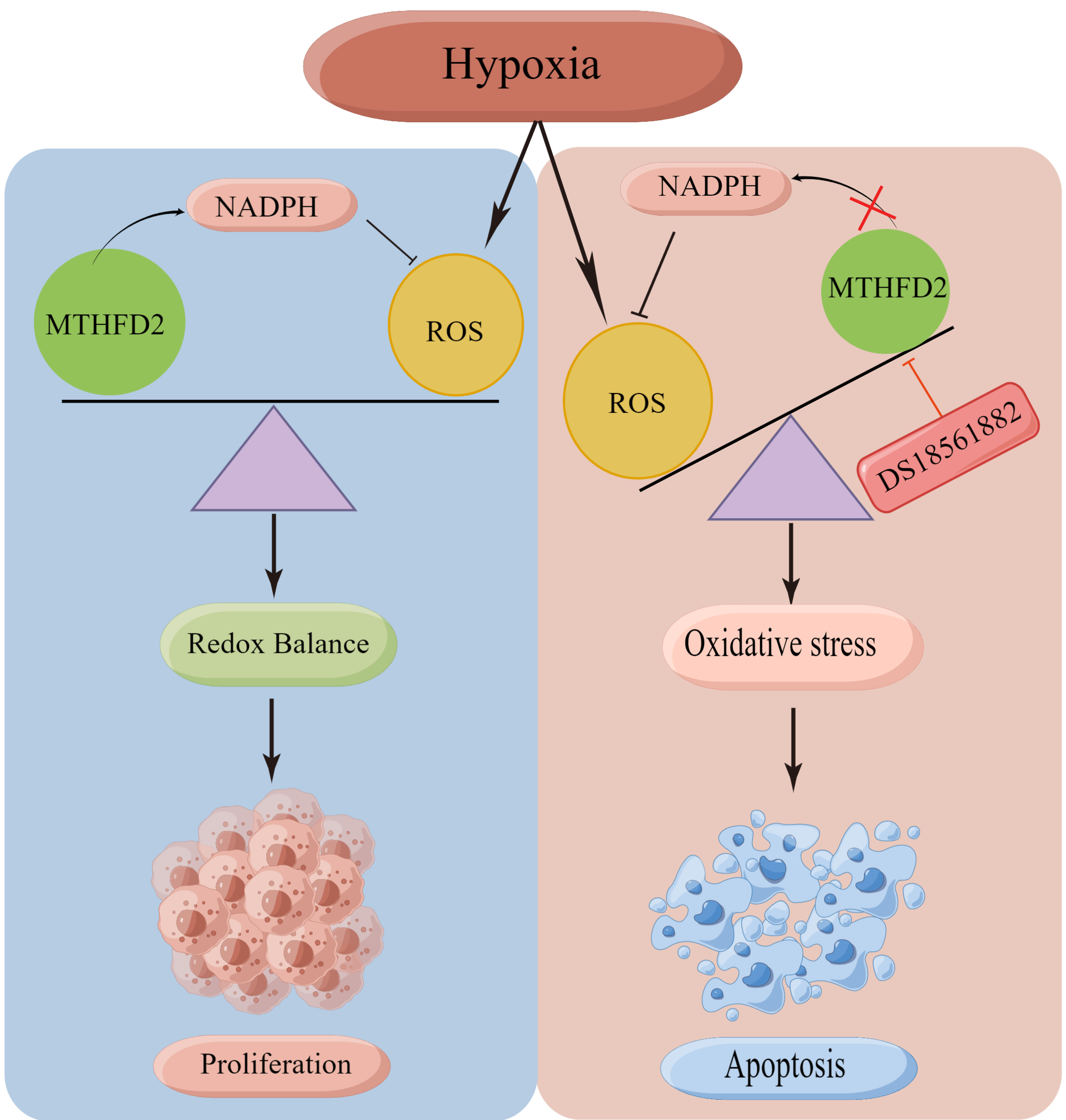

Supplement: graphical abstract.tif [file YRER_A_2345455_SM1379.tif]
